# Remember the past, plan for the future: How interactions between risk perception and behavior during the COVID-19 pandemic can inform future Canadian public health policy

**DOI:** 10.3389/fpubh.2022.784955

**Published:** 2022-08-03

**Authors:** Moira A. Law, Jonathan M. P. Wilbiks, Sean P. Roach, Lisa A. Best

**Affiliations:** Department of Psychology, University of New Brunswick Saint John, Saint John, NB, Canada

**Keywords:** COVID-19, public health policy, collective behavior, behavioral adherence, public mental health

## Abstract

The ongoing COVID-19 pandemic necessitated the implementation of numerous temporary public health policies, including social distancing, masking, and movement limitations. These types of measures require most citizens to follow them to be effective at a population level. This study examined population adherence to emergency public health measures using early data collected in the Spring of 2020, when all Canadian jurisdictions were under relatively strict measures. In total, 1,369 participants completed an online questionnaire package to assess adherence, perceptions of government response, and perceptions of COVID-19 risk. Results indicated that most Canadians were pleased with the government's handling of the early phases of the pandemic and immediately engaged new public health mandates. Willingness to change behaviors was unrelated to satisfaction with the government response. Similarly, behavioral adherence was also unrelated to satisfaction with government, or personal risk perceptions; however, adherence to public health guidelines was related to elevated psychological distress. As the pandemic continues, public health officials must balance the mental health of the population with the physical health concerns posed by COVID-19 when applying public health mandates.

## Introduction

The current COVID-19 pandemic has been a novel situation with many unknowns, including how individuals would respond to the pandemic itself and how they would respond to associated public health recommendations, guidelines, and policies. Although public health measures vary in their effectiveness and can have effects on both physical and mental health (1, 2), adherence is typically high in emergency situations; for example, Tracy et al. (3) reported that when quarantine is required, the public generally supports governmental decisions. Nonetheless, because even the implementation of less restrictive measures can lead to distress among Canadian populations (4), public health authorities must strike a balance between physical and mental health risks. An understanding of how individuals perceive government responses to pandemics and, importantly, how that relates to their adherence with public health policies is vital. Although preventative regulations focus on preventing transmission of the SARS-CoV-2 virus, population-level adherence to such measures can be influenced by several factors that vary widely depending on global location; these factors include psychological wellbeing, personal risk perception, as well as impressions of government competence and health care system capacity. For instance, many countries in the Global South did not have the economic and organizational capacity to swiftly respond to the current pandemic, hence their citizenry's initial behavioral adherence reflected their vulnerable circumstances, such as poor employment conditions, as well as perceptions of government and interpersonal characteristics, including risk perception (5–7).

Although perceptions of specific health measures influence adherence to behaviors (8, 9), the factors influencing this relationship are not fully clear. Although increased risk perception, fear, and anxiety are associated with preventive actions, including frequent handwashing, social distancing, and self-isolation (2, 10, 11), higher adherence is not always associated with greater risk of disease spread. For example, during the 2009–2010 H1N1 outbreak of influenza in Hong Kong, although the risk of individuals contracting disease was low there was a widespread acceptance of avoidance behaviors (12).

During the H1N1 epidemic in Beijing, Xu and Peng (13) used a longitudinal design to examine people's perceptions of the disease and their behaviors at various stages of the pandemic. During the pre-pandemic phase, behaviors recommended by public health officials to reduce transmission were inversely related to personal risk perceptions, such that those persons with higher estimates of their own risks associated with contracting the disease, were less likely to engage in the recommended behaviors. During the rising phase, there was a positive relationship; individuals who believed they were at risk of infection were *more* likely to engage in such behaviors. Finally, at the peak of the pandemic, the association between risk perception and adherence was less clear; social distancing was positively associated with perceived risk, but hand hygiene was not. Thus, further investigation is required to clarify the factors that govern adherence to public health policies in emergency situations.

The purpose of this study was to examine the relationships between risk perception, psychological distress, perceptions of government performance, and behavioral adherence to public health directives during the COVID-19 pandemic in Canada, March 31–April 15, 2020. Research during the COVID-19 pandemic has indicated that women exhibit higher adherence to public health measures (5, 14), higher COVID-19 risk perceptions (15) and more psychological distress (16). Hence, we also surveyed sex difference among the observed associations.

## Method

### Participants

In total, 297 males and 1,072 females completed an online questionnaire package. The mean age of females was slightly lower than that of the males M_age_ = 40.61, *SD* = 14.76 vs. 43.48, *SD* = 17.29; *t*_(1,367)_ = 2.85*, p* = 0.01. In addition, 12 participants identified as neither male nor female and these participants were significantly younger, M_age_ = 34.17, *SD* = 15.35. Most participants reported that they were Caucasian, *n* = 1,295; 93.5%; 2.5% of participants reported that they were East Asian or Asian and ~1% of participants reported that they were Black. Most participants were currently enrolled (*n* = 257) in or completed (*n* = 551) post-secondary education programs, with 464 participants who were enrolled in or had completed a graduate or professional program.

### Materials

Behavioral adherence was measured using seven items rated on a 4-point Likert scale ranging from “always” to “never.” Items assessed specific aspects of social distancing (e.g., I avoid crowded places) and hygiene behaviors (e.g., hand washing). For each item, participants also indicated (yes or no) if their behavior had changed because of COVID-19, with lower scores indicating higher adherence. The Cronbach's α was 0.76 and 0.64 for the adherence behaviors and change items, respectively.

Risk perception was evaluated using a five-item questionnaire rated on a five-point Likert scale (1 = strongly disagree) to assess perception of risk related to the virus, e.g., “I believe there is a high risk of death if someone contracted COVID-19.” This measure had adequate reliability, with Cronbach's α = 0.72. COVID-19 Worry was assessed using five questions adapted from Lau et al. (17). Participants used a 5-point Likert scale (1 = strongly disagree) to rate their panic, depression, and emotional stability as well as the degree to which they were worried about their personal and family safety. The reliability of this measure was high, Cronbach's α = 0.82.

Public perception of government performance was assessed using a seven-item self-report questionnaire. Based on a scale from 1 to 10, with 5 considered a passing grade, participants assessed government performance reporting on how satisfied they were with the measures being taken to prevent the spread of the virus, the timeliness of measures, and the effectiveness of implemented measures.

### Procedure

Data collection for this study took place between March 31 and April 15, 2020 when strict social distancing regulations were implemented in all Canadian provinces and territories. Participants were recruited from social media sites (i.e., Facebook, Twitter) and were directed to an online survey platform (Qualtrics). We recruited broadly and our questionnaire did not include questions to examine individual history of COVID-19 infection. After providing informed consent and answering basic demographic questions, participants completed the randomized questionnaire package. Questionnaire completion took ~12 min. This study was reviewed and approved by the University of New Brunswick Research Ethics Board.

### Data analysis strategy

SPSS V. 28 was used for data analysis. Prior to data analysis, data conditioning was conducted to ensure there were no out-of-range values or missing data. The assumptions underlying the statistical tests were examined. Correlational analyses were used to examine the associations between risk perception, psychological distress, perceptions of government performance, and behavioral adherence. *T*-tests were used to examine specific gender differences and a mixed model analysis of variance (ANOVA) was used to examine adherence as a function of gender and education.

## Results

Canadians exhibited overall satisfaction with their government, with responses on the government performance questionnaire indicating a higher than acceptable rating (M = 5.42, *SD* = 1.15) (see Table 1 for descriptive statistics and correlations between study variables). A correlational analysis indicated that mean perception of Government Performance was significantly associated with lower overall Risk Perception, *r*_(901)_ = −0.12*, p* < 0.001, as well as perceived Personal, *r*_(900)_ = −0.09, *p* = 0.005, and Family, *r*_(899)_ = −0.10, *p* = 0.002, risk of contraction. Mean Risk Perception was associated with adherence to Personal Hygiene guidelines, *r*_(892)_ = −0.09, *p* = 0.01, but not with adherence to Social Distancing guidelines, *r*_(890)_ = −0.03, *p* = 0.40. Correlational analyses were also conducted to determine if negative psychological outcomes were related to adherence to government directives for social distancing and personal hygiene. The correlations between overall COVID-19 Worry and Social Distancing, *r*_(896)_ = −0.12, *p* = 0.001, and Personal Hygiene, *r*_(896)_ = −0.12, *p* < 0.001, indicated that individuals who experienced more COVID-19 Distress were more likely to follow guidelines.

**Table 1:** Mean (standard deviation) and correlations between variables of interest.

	**Risk perception**	**COVID-19 worry**	**Government performance**	**Adherence: social avoidance**	**Social avoidance change**	**Adherence: hygiene**	**Hygiene change**
**Mean (sd)**	3.08 (0.76)	3.41 (0.91)	5.42 (1.15)	3.71 (1.40)	0.76 (0.23)	1.26 (0.49)	0.81 (0.40)
Risk perception		0.444^***^	−0.125^***^	−0.014	0.054	−0.086^**^	0.006
COVID-19 worry			−0.067^*^	−0.109^**^	0.156^***^	−0.118^***^	0.142^***^
Government performance				−0.035	0.022	0.020	0.025
Social avoidance					−0.001	0.285^***^	−0.010
Social avoidance Change						−0.011	0.356^***^
Hygiene							0.100^**^

* p < 0.05,

** p < 0.01,

*** p < 0.001.

Social Avoidance and Hygiene were reverse scored, such that lower numbers indicated increased adherence.

**Figure 1:**
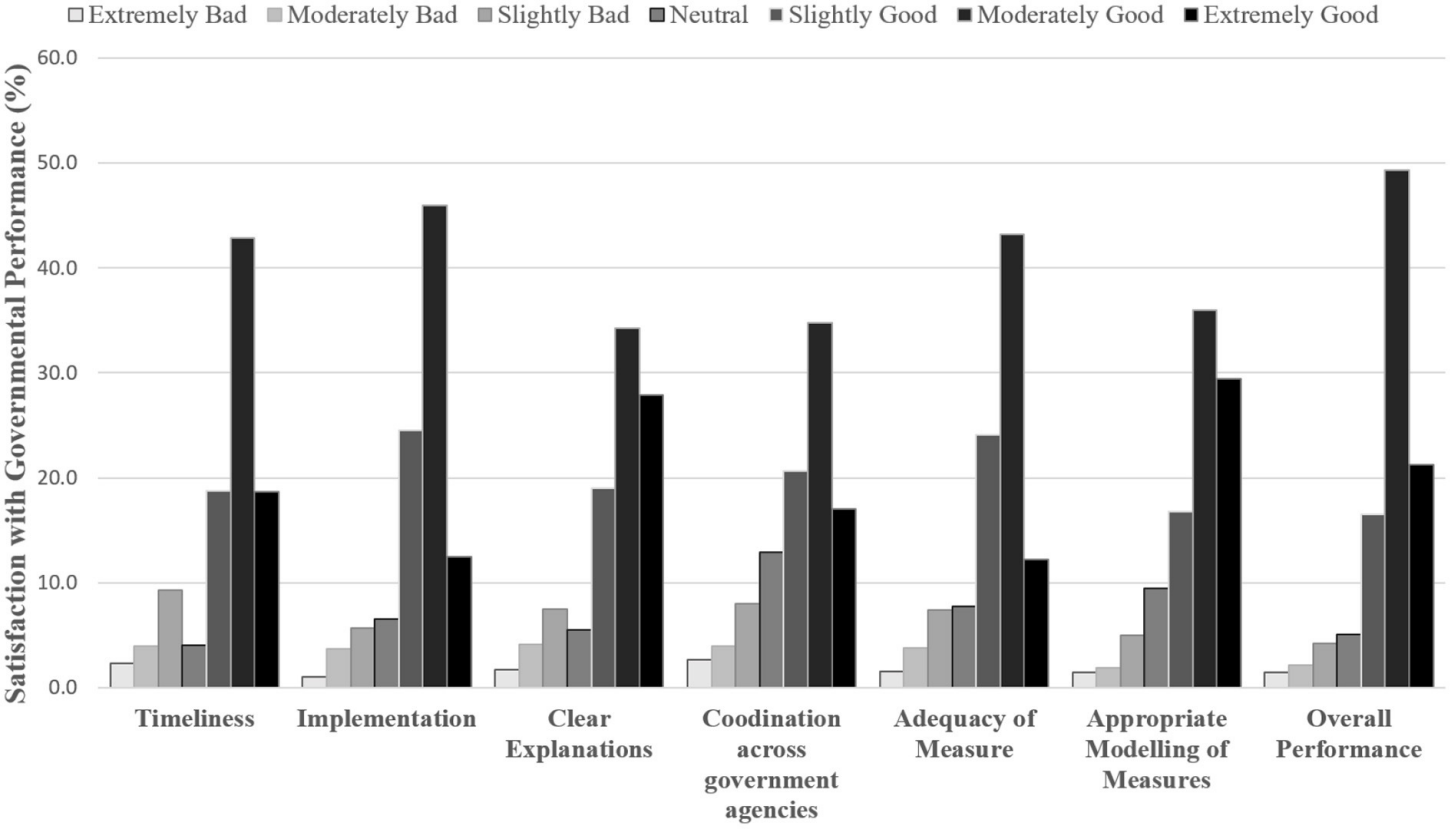
Satisfaction of participants with government measures (N = 1,386).

Mean satisfaction with Government Performance was 5.42 (*SD* = 1.15); <20% of participants rated Government Performance negatively (Figure 1). Although there was no difference in satisfaction among males and females (*M* = 5.33 and 5.45, respectively; independent samples *t*-test: *t*_(1,110)_ = 1.39*, p* = 0.17), there was a statistically significant correlation between age and satisfaction with Government Performance, *r*_(1,382)_ = 0.102, *p* < 0.001, indicating that older participants reported higher satisfaction than did younger participants.

In addition to being satisfied with the governmental response, respondents were optimistic about the ability of the local health care system to manage the pandemic. Respondents reported that their local health system had sufficient space, *M* = 3.82, *SD* = 1.12, enough medical personnel, *M* = 3.77, *SD* = 1.10, and adequate personal protective equipment, *M* = 4.01, *SD* = 1.04. Further, participants believed that the Canadian government would be able to control the current pandemic, *M* = 3.14, *SD* = 1.04, although there was less confidence in the ability to manage a large scale COVID-19 outbreak, *M* = 3.02, *SD* = 1.10.

Virtually all participants reported that their behaviors had changed due to government directives and reflected social distancing and personal hygiene recommendations. Participants reported that their social distancing and personal hygiene behaviors changed in response to the pandemic (average reported change was 85.5%). Although there were sex differences in social distancing, the degree of behavioral change in response to COVID-19 was similar for males and females. The degree of behavior change varied across measures, with greater change for social distancing measures (e.g., respecting social distancing guidelines) and less change for food sharing, likely because participants avoided food sharing prior to the pandemic. Further, although there were differences in how satisfied participants were with specific governmental responses, dissatisfaction with the government did not affect the behavioral changes associated with preventing COVID-19.

Compared to males, females were more likely to comply with social distancing, *t*_(1,114)_ = 3.08*, p* = 0.002, and hygiene, *t*_(1,111)_ = 3.10*, p* = 0.002, guidelines. Further, correlational analyses indicated that older individuals were more likely to be satisfied with overall government performance, *r*_(1,110)_ = 0.10, *p* < 0.001, and adhere to social distancing, *r*_(1,113)_ = 0.13, *p* < 0.001, guidelines. To control for potential effects of age, a partial correlational analysis was used to examine the association perceived government performance and compliance with recommendations in the overall sample. The partial correlations between perceived satisfaction with government performance and overall compliance were not significantly associated with social distancing, *r*_(873)_ = −0.036, *p* = 0.29, or personal hygiene, *r*_(873)_ = 0.04, *p* = 0.26.

To examine specific differences in adherence to government directions as a function of demographic variables a 2 (sex) × 2 (education: university vs. no university) × 2 (measure type: social distancing, hygiene) mixed model analysis was conducted (see Table 2). There were statistically significant main effects of sex, *F*_(1,1106)_ = 16.54, *p* < 0.001, with females exhibiting greater overall compliance, and measure type, *F*_(1,1,106)_ = 50.65, *p* < 0.001, with participants reporting higher compliance with social distancing guidelines than with personal hygiene.

**Table 2:** Mean differences (and standard deviations) in adherence of government guidelines as a function of sex and education.

	**No university education**			**University education**		
**I avoid..**.	**Males**	**Females**	**Total**	**Males**	**Females**	**Total**
…crowded places (i.e., to practice social distancing).	1.31 (0.56)	1.17 (0.42)	1.20 (0.46)	1.18 (0.46)	1.15 (0.41)	1.15 (0.42)
…going out unless necessary.	1.38 (0.63)	1.28 (0.55)	1.30 (0.57)	1.41 (0.63)	1.25 (0.54)	1.28 (0.57)
…shaking hands.	1.67 (1.00)	1.38 (0.78)	1.45 (0.84)	1.62 (0.97)	1.46 (0.83)	1.49 (0.86)
…sharing my food and drinks.	1.52 (0.76)	1.49 (0.72)	1.49 (0.73)	1.46 (0.66)	1.46 (0.67)	1.46 (0.68)
…sitting directly next to someone.	1.71 (0.88)	1.60 (0.78)	1.62 (0.81)	1.71 (0.87)	1.59 (0.82)	1.62 (0.83)
Social avoidance mean	1.52 (0.52)	1.38 (0.48)	1.41 (0.49)	1.48 (0.51)	1.38 (0.48)	1.40 (0.49)
I practice proper hygiene and regularly wash hands, minimum 20 s.	1.34 (0.54)	1.19 (0.42)	1.23 (0.45)	1.36 (0.55)	1.26 (0.51)	1.28 (0.52)

*Adherence was rated on a 1 (always) to 4 (never) scale, with lower scores indicating higher adherence to guidelines*.

## Discussion

During the initial stages of the COVID-19 pandemic, between March 31 and April 15, 2020, most Canadians (80%) surveyed were satisfied with the performance of their government. Notwithstanding limitations of generalizability due to this survey being launched on social media platforms this high approval rating was similar to a global sample of 25,992 adults aged 18–74 years surveyed during the week of April 23–26th that reported comparable satisfaction rates of their government response by Canadians (81%), Indians (87%), and Australians (84%); and much higher than Japanese (31%), Russians (38%) and French (43%) citizens (18). The absence of a gender difference with respect to government satisfaction was surprising given the documented disparities women have experienced during this state of emergency in terms of caregiving responsibilities (19), perceived risks to family members (4) and employment disruptions (20). Perhaps the anticipated gender differences would have emerged if data collection was longer than 2 weeks and later in the pandemic (21, 22). Another notable limitation of this study is the high number of female respondents, 78% (*n* = 1,072), reducing the generalizability of our findings and marking the need for replication with more representative samples. Higher female participation may have led to higher rates of reported psychological distress, behavioral adherence, and risk perceptions in this study (5, 22–26).

Most participants (85%) reported immediately changing their behaviors due to the pandemic, exhibiting widespread adherence to social distancing and personal hygiene recommendations. Interestingly, although there was no sex difference in governmental satisfaction, females adhered more closely than males to all public health policies from March 31–April 15, 2020. These findings are similar to studies conducted in March 21–26, 2020 (14) and March to December 2020 (5) and align with an earlier study that found women had higher risk perceptions for family members than for themselves during the earliest days of this outbreak; it was for the safety of loved ones rather than themselves that motivated behavioral changes (4). One year later, distressed concern for loved ones continued (23, 27) and may support public health maintaining a focus on compassionate messaging to motivate adherence behaviors as the pandemic continues (24, 25).

A notable limitation of this study is the high proportion of respondents with some or completed post-secondary education limiting the conclusions that can be drawn for the broader population. Unlike other studies that found higher adherence was related to higher levels of education (26, 28, 29), the current results did not indicate an association between levels of education and adherence to public health guidelines suggesting initial adherence may have been primarily motivated by emotional response rather than reason (30).

Global fear quickly rose as mainstream and social media's growing coverage on the spread of the SARS-CoV-2 virus abroad may have been affecting Canadian citizens well before March 2020 when the country went into lockdown (31). The current pandemic led to unprecedented connectivity to sources of information that were both reliable, e.g., public health briefings, and unreliable, e.g., social media, often to the detriment of the public's wellbeing (32, 33). Misinformation became mainstream (34) and even peer-reviewed scientific publications that generated initial overestimations of infection mortality rates contributed to the public's mounting angst (35). The interplay of social contagion via social media and disease spread may have been contributing to growing fear (36) that directed early adherence behaviors measured in this study and detected in other studies in the same time frame (4, 30, 33) neutralizing any effects of education and critical thinking early at this stage in this pandemic. Later studies provided compelling evidence that education is a moderator of employment conditions that affords more choice in social distancing requirements (37, 38), giving rise to a significant disparity between the “laptop class” and front line workers (39) that may require government and public health coordination when considering social mitigation in the future, i.e., enhanced social assistance.

Older Canadians reported higher satisfaction and behavioral adherence with the government's early response to the pandemic, perhaps reflecting their knowledge of being at greater risks for adverse COVID outcomes (31) and their relief to see governmental responses unfolding quickly and in a unified manner (40). With most Canadian COVID-19 deaths reported among seniors (41) since this early data was collected it is expected adherence in this population will remain high. Strikingly, there was no relationship between government satisfaction and adherence with public health guidelines, which highlights the need for a better understanding of the factors and context influencing adherence behaviors that are vital to successful pandemic mitigation.

Individuals with an overall lower perception of personal and family risk assessed the government's response more positively, suggesting public health officials would be wise to deescalate the public's personal risk perceptions by continuing to provide timely and accurate information during future outbreaks (42). Surprisingly, overall risk perception was not associated with social distancing behaviors, e.g., standing 2 m apart, but significantly related to personal hygiene, e.g., hand washing directives. This was the opposite of findings from the peak of the H1N1 outbreak, in which social distancing was positively associated with perceived risk but hand hygiene was not (13). This is interesting as hygiene behaviors, such as hand washing (43, 44) and sneezing into elbow (45), have a well-established evidence base compared with social distancing behaviors (46). Future pandemic investigations should consider the extrinsic and intrinsic motivation of adherence behaviors across genders, different age groups, and those with elevated risk perceptions.

Finally, and not surprisingly, individuals who experienced elevated levels of worry and distress were more likely to adhere to public health guidelines and report that their behaviors changed in response to the current pandemic as noted in previous pandemics (2, 10, 11). Despite these results, public health officials should be reminded that excessive and prolonged stress interferes with adherence (47–50) and mental health professionals have been sounding the alarm on elevated mental health conditions as the pandemic has progressed (51–54). In addition to guidelines designed to curtail disease spread as new variants of the SARS-CoV-2 virus emerge, managing risk perceptions for various subpopulations, and incorporating broader definitions of health that supersede single factor analysis, e.g., physical health (40, 41) need to become integrated into public health management plans.

Canadian policy makers need to be cognizant of co-operating within international frameworks that will serve Canadians and other countries well and remain aware of issues regarding vaccine availability, systemic disadvantages, and daily individual struggles that are commonplace in other countries (55). Moderate policies that are not too strong or too weak optimize desired health outcomes (56). For instance, policies that reduce social contacts to a moderate level and avoid full lockdowns may achieve outcomes that protect the healthcare system and avoid economic consequences (57) while avoiding severe conditions that exacerbate psychological distress. This relationship between psychological distress and adherence to public health directives warrants continued monitoring as the effects of prolonged mitigation may evolve into serious pathology and adherence behaviors deteriorate due to psychological fatigue. In short, the secondary impacts of social mitigation, such as deterioration in mental health (4) and economic repercussions (46, 57) must be heavily factored into public health plans as the country continues to move forward.

## Data availability statement

The raw data supporting the conclusions of this article will be made available by the authors, without undue reservation.

## Ethics statement

The studies involving human participants were reviewed and approved by Research Ethics Board at the University of New Brunswick Saint John. The patients/participants provided their written informed consent to participate in this study.

## Author contributions

LB analyzed the data and created figures. ML wrote the initial draft of the manuscript. JW, SR, and LB revised the manuscript. All authors conceptualized the paper, designed the method, and collected data and have reviewed and approved the final submission.

## Conflict of interest

The authors declare that the research was conducted in the absence of any commercial or financial relationships that could be construed as a potential conflict of interest.

## Publisher's note

All claims expressed in this article are solely those of the authors and do not necessarily represent those of their affiliated organizations, or those of the publisher, the editors and the reviewers. Any product that may be evaluated in this article, or claim that may be made by its manufacturer, is not guaranteed or endorsed by the publisher.
